# Projected Changes in Yield and Water Use Efficiency of Cold-Region Rice and the Role of CO_2_ Under Climate Change

**DOI:** 10.3390/plants15111625

**Published:** 2026-05-26

**Authors:** Zhinan Li, Ying Liu, Tangzhe Nie, Xingtao Xiao, Hang Guo, Tianyi Wang, Yu Han

**Affiliations:** 1 School of Water Conservancy and Electric Power, Heilongjiang University, Harbin 150080, China; lizhinan293610@gmail.com (Z.L.); 2025110@hlju.edu.cn (H.G.); 2025069@hlju.edu.cn (T.W.); 2 Heilongjiang Provincial Hydrology and Water Resources Center, Harbin 150001, China; 3 School of Water Conservancy and Civil Engineering, Northeast Agricultural University, Harbin 150030, China; b220101004@neau.edu.cn

**Keywords:** climate change, rice yield, crop evapotranspiration, water use efficiency, AquaCrop model, SSP scenarios

## Abstract

Climate change is reshaping yield formation and water use in cold-region rice production through rising air temperatures, altered precipitation patterns, and increasing atmospheric CO_2_ concentrations. However, the responses of yield, crop evapotranspiration (*ET_c_*), and water use efficiency (*WUE*) to climate forcing and elevated CO_2_ remain insufficiently quantified for cold-region rice systems in Northeast China. This study simulated changes in rice yield, *ET_c_* and *WUE* during the 2030s–2090s relative to the 2000–2020 baseline period under the SSP1-2.6, SSP2-4.5, and SSP5-8.5 scenarios at 10 agro-meteorological stations in Heilongjiang Province. Simulations were conducted using the AquaCrop model driven by CMIP6 multi-model climate data, and the contribution of elevated CO_2_ was quantified by comparing the rising-CO_2_ and fixed-CO_2_ treatments. The results showed that under SSP5-8.5, the maximum air temperature in the 2090s is projected to increase by 5~6 °C relative to the baseline period, while precipitation is projected to range from −10% to 20%. Compared with the fixed-CO_2_ treatment, rice yield under the rising-CO_2_ treatment is projected to increase by 18.70%. Although *ET_c_* showed an overall increasing trend, rising CO_2_ attenuated its increase. Under SSP5-8.5 in the 2090s, *ET_c_* increased by only 2.70% under rising-CO_2_ treatment, compared with 11.61% under fixed-CO_2_ treatment. As a result of increased yield and *ET_c_*, the *WUE* improved by 15.42% and 14.28% under SSP2-4.5 and SSP5-8.5, respectively, in the 2090s, whereas it remained below the baseline level under the scenarios without CO_2_ effects. These findings indicate that rising CO_2_ may enhance yield and moderate *ET_c_* increases, thereby providing useful information for regional grain-yield assessment, agricultural water-resource evaluation, and climate-change adaptation planning.

## 1. Introduction

Climate change is reshaping the thermal, hydrological, and atmospheric conditions on which agricultural yield depends and has already affected global food and water security [1]. Even when climatic adaptation measures are considered, climate change will continue to influence the yield of major food crops worldwide, and these impacts may emerge earlier in major grain-producing regions than previously expected [2,3]. Future agricultural yield will face not only greater variability in crop yields but also changes in irrigation demand, shifts in agricultural water-use processes, and increasing pressure on regional water allocation [4,5,6]. For agricultural systems that depend on field water management, jointly examining changes in crop yield and crop evapotranspiration (*ET_c_*) under climate change is therefore essential for safeguarding food security, improving water use efficiency (*WUE*), and supporting sustainable agricultural development [1,4,5,6].

Many studies have examined the effects of changes in air temperature, precipitation, and atmospheric CO_2_ concentration on crop yield [7,8,9,10]. In general, air temperature affects crop growth, dry-matter accumulation, and yield formation [7,8,10], whereas precipitation influences yield by altering soil moisture conditions and the seasonal distribution of rainfall, with the magnitude of the effect moderated by factors [7,11,12]. Elevated CO_2_ concentration usually enhances photosynthesis and improves resource-use efficiency, and this effect is often more pronounced in C3 crops, including rice [13,14]. However, the effects of these three factors are not simply additive. Elevated CO_2_ may promote photosynthesis and partly alleviate water stress. However, changes in air temperature may offset these benefits by shortening the growing period and intensifying heat stress. Increased precipitation does not necessarily lead to higher yield [9,12,14,15]. Crop-yield responses to future climate change therefore reflect the combined effects of air temperature, precipitation and CO_2_ concentration [7,9,16]. Although this general pattern is well established, substantial uncertainty remains regarding the relative importance, direction and magnitude of these effects under future climate across spatial scales, emission pathways and model settings [7,9,16].

Crop evapotranspiration (*ET_c_*) directly reflects crop water use during the growing season and is closely linked to irrigation demand and regional water-resource allocation [17,18]. Rising air temperature generally increases atmospheric evaporative demand and thus tends to increase *ET_c_* [17,19]. Changes in precipitation modify water availability in cropland and thereby influence soil evaporation and crop transpiration [17,19]. At the same time, rising-CO_2_ concentrations may alter *ET_c_* through their effects on stomatal conductance and canopy water use [13,20]. Compared with yield-focused studies, integrated assessments that examine *ET_c_* together with yield and *WUE* under explicit rising-CO_2_ and fixed-CO_2_ treatments remain limited, particularly for cold-region rice systems. Studies that simultaneously consider air temperature, precipitation and CO_2_ when analyzing *ET_c_* remain limited, and it is still unclear whether changes in yield are accompanied by similar changes in *ET_c_* [7,18,21]. Clarifying how *ET_c_* responds to these climatic drivers is therefore important for understanding future crop water demand and improving agricultural water management under climate change [7,18].

The *WUE* is defined at the crop-season scale as grain yield per unit of seasonal *ET_c_*. This differs from transpiration efficiency, which usually refers to biomass or yield produced per unit of crop transpiration and excludes non-productive soil evaporation [22,23]. Changes in *WUE* depend on the relative direction and magnitude of changes in yield and *ET_c_* [22,23]. For example, rising CO_2_ may simultaneously increase yield and reduce *ET_c_*, thereby improving *WUE*, whereas increased air temperature may weaken these gains by increasing evaporative demand and altering crop growth processes; changes in precipitation further modify these responses across regions and scenarios [20,22,24]. *WUE* is therefore not a simple extension of changes in yield or *ET_c_*, but a combined outcome of multiple climatic drivers [22,23,24]. This question is especially relevant across different SSP pathways. Contrasting air temperature, precipitation, and CO_2_ concentration changes may produce substantially different combinations of yield and *ET_c_* responses. Existing studies frequently discuss either yield or water use, but fewer place yield, *ET_c_*, and *WUE* within the same analytical framework and explicitly quantify the contribution of CO_2_ [18,21,23]. Therefore, assessing *WUE* is important not merely because integrated analyses remain limited, but because it is central to understanding whether future climate scenarios will favor a more productive and water-efficient rice production system.

Heilongjiang Province in Northeast China is a typical cold-region rice-growing area and one of the most important rice-producing regions in China. Rice yield in this province is highly sensitive to growing-season thermal conditions, and changes in air temperature directly affect phenological development and yield formation [24,25,26,27]. Meanwhile, agricultural water use is shaped by the seasonal distribution of precipitation, the high water requirement of paddy fields, and local water availability. In addition, increasing pressure on regional water resources has raised concern over the sustainability of rice production [28,29,30]. Heilongjiang Province therefore provides a suitable setting in which to study the combined effects of air temperature, precipitation, and CO_2_ on rice yield, *ET_c_*, and *WUE* under climate change [27,31].

Previous crop–climate modeling studies have substantially advanced understanding of climate impacts on rice production, but many have focused primarily on yield responses or irrigation water demand in warmer rice-growing regions. Fewer studies have jointly evaluated yield, *ET_c_*, and *ET_c_*-based *WUE* under explicit rising CO_2_ and fixed-CO_2_ treatments at the station scale in cold-region rice systems. We hypothesized that: (i) moderate warming would partly relieve low-temperature constraints on rice growth in Heilongjiang Province, although this benefit may weaken under late-century high-emission warming; (ii) rising CO_2_ would increase yield while moderating the increase in *ET_c_* through physiological regulation of transpiration; and (iii) projected *WUE* changes would depend mainly on the balance between CO_2_-induced yield enhancement and changes in seasonal *ET_c_*.

The present study therefore contributes by combining AquaCrop with CMIP6 multi-model climate projections to examine the coupled yield and water response of rice across 10 agro-meteorological stations in Heilongjiang Province, a major cold-region rice-producing area of Northeast China. This study aims to: (1) characterize future changes in growing-season air temperature and precipitation under SSP1-2.6, SSP2-4.5 and SSP5-8.5; (2) clarify projected changes in rice yield, *ET_c_*, and *WUE*; and (3) quantify the contribution of CO_2_ to changes in these indicators. By jointly examining crop yield and agricultural water use, this study provides a basis for understanding climate-change impacts in cold-region rice-growing areas and for supporting adaptation planning for rice yield and agricultural water management in Heilongjiang Province.

## 2. Results

### 2.1. Projected Climate Change

#### 2.1.1. Relative Changes in Air Temperature

**Figure 1:**
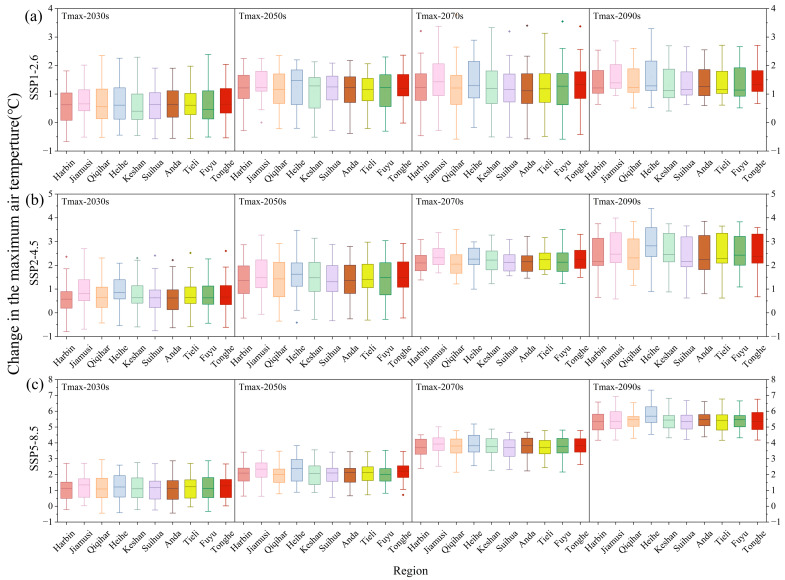
Relative changes in maximum air temperature during the rice-growing season in the study area from the 2030s to the 2090s compared to the baseline period under (**a**) SSP1-2.6, (**b**) SSP2-4.5, and (**c**) SSP5-8.5. *T_max_* represents the maximum air temperature.

**Figure 2:**
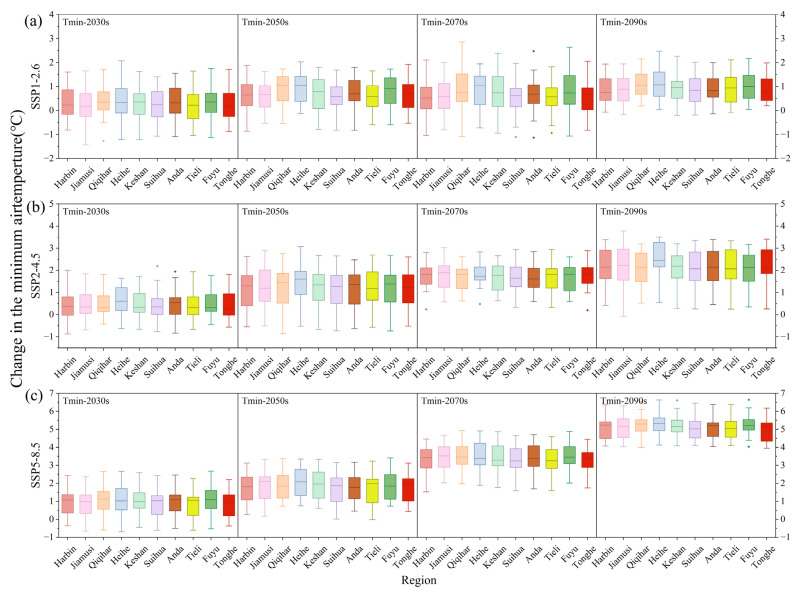
Relative changes in minimum air temperature during the rice-growing season in the study area from the 2030s to the 2090s compared to the baseline period under (**a**) SSP1-2.6, (**b**) SSP2-4.5, and (**c**) SSP5-8.5. *T_min_* represents the minimum air temperature.

Relative maximum air temperature (*T_max_*) during the rice-growing season increases under 3 SSPs (Figure 1). Under the SSP1-2.6, the median increase in *T_max_* at 10 stations between 2030 and 2090 is concentrated in the range of 0.40~1.39 °C, with little variation across different time periods. Under SSP2-4.5, the median increase grows gradually from 0.57~0.84 °C in the 2030s to 2.16~2.82 °C in the 2090s. Under SSP5-8.5, the increase is much larger, rising from 1.10~1.34 °C in the 2030s to 5.34~5.69 °C in the 2090s. Differences among the three scenarios widen over time, and the extent of air temperature increase differs across stations. Relative minimum air temperature (*T_min_*) shows a similar pattern (Figure 2). Under SSP1-2.6, the median increase in *T_min_* across all time periods is about 0.18~1.07 °C; under SSP2-4.5, it rises from 0.26~0.61 °C in the 2030s to 2.07~2.43 °C in the 2090s; and under SSP5-8.5, the median increase reaches 5.02~5.33 °C by the 2090s.

#### 2.1.2. Relative Changes in Precipitation

**Figure 3:**
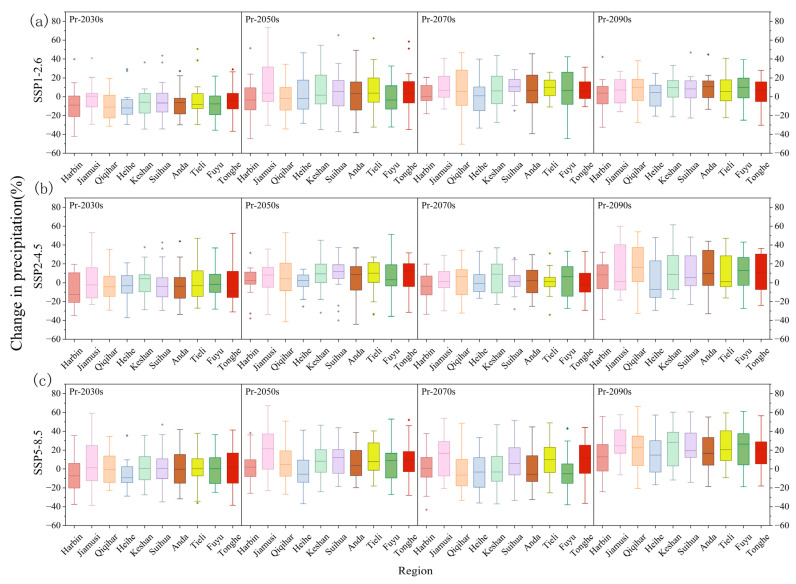
Relative changes in precipitation during the rice-growing season in the study area from the 2030s to the 2090s compared to the baseline period under (**a**) SSP1-2.6, (**b**) SSP2-4.5, and (**c**) SSP5-8.5. *Pr* represents the precipitation.

Relative changes in precipitation (*P_r_*) during the rice-growing season vary across three SSPs (Figure 3). The boxplots generally show wide interquartile ranges at most stations, indicating considerable variability and clear inter-station differences. Across the four periods, the median changes range from −12.63% to +28.29%. Under SSP1-2.6, the median precipitation change at most stations ranges from −11.94% to 0.64% in the 2030s. The median then increases in the 2050s and 2070s, with most values ranging from −3.52% to 10.61%. Under SSP2-4.5, median precipitation changes are generally positive in most stations. From the 2050s to the 2090s, the median increase at most stations ranges from 2.18% to 16.36%. Under SSP5-8.5, the median precipitation change shows a slight increase, and the overall median increase is approximately −1.39% to 21.66%.

#### 2.1.3. Changes in CO_2_ Concentration

**Figure 4:**
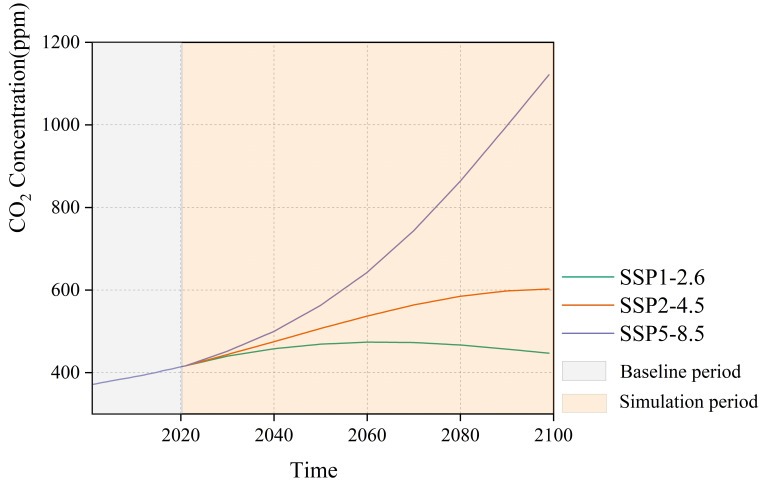
Concentration changes in atmospheric CO_2_ concentration in the study area under different SSPs from the 2030s to the 2090s.

The concentration changes in atmospheric CO_2_ concentration differ among the three SSPs (Figure 4). Under SSP1-2.6, CO_2_ peaks at 467.90 ppm in the 2050s and then stabilizes or declines slightly to 456.76 ppm by the 2090s. Under SSP2-4.5, CO_2_ continues to rise and reaches 596.11 ppm by the 2090s. Under SSP5-8.5, CO_2_ increases much more rapidly, rising from 456.31 ppm in the 2030s to 998.71 ppm in the 2090s, nearly a twofold increase. Across the three scenarios, CO_2_ concentration ranges from 439 to 456 ppm in the 2030s, but widen sharply by the 2090s, with SSP5-8.5 exceeding SSP1-2.6 by more than 540 ppm.

### 2.2. Calibration and Validation

The AquaCrop model was calibrated and validated independently for the study area using observed data. For seasonal crop evapotranspiration (*ET_c_*) and rice yield (*Y*), the model showed low error and high agreement during both the calibration and validation periods, with NRMSE values below 10% and EF and d values close to 1 (Table 1). These results indicate that the model reproduced *ET_c_* satisfactorily.

**Figure 5:**
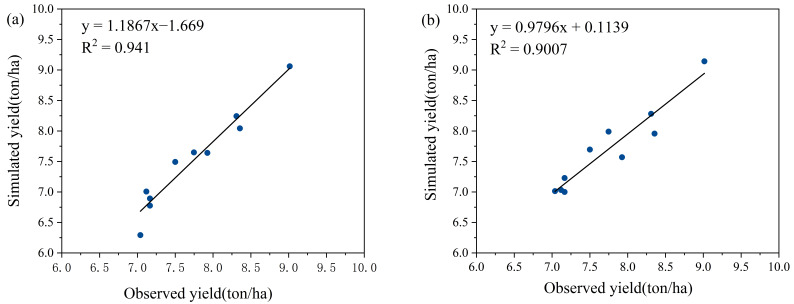
Comparison between observed and simulated rice yield during the (**a**) calibration and (**b**) validation periods.

For rice yield, the agreement between simulated and observed values was also strong, as shown by the close correspondence between the two datasets in Figure 5. Overall, the calibration and validation results demonstrate that the AquaCrop model can reasonably reproduce both crop water consumption and yield formation in the study area, and is therefore suitable for subsequent simulations of rice yield, *ET_c_*, and *WUE* under future climate scenarios.

### 2.3. Yield and Net CO_2_ Contribution

#### 2.3.1. Relative Changes in Yield

**Figure 6:**
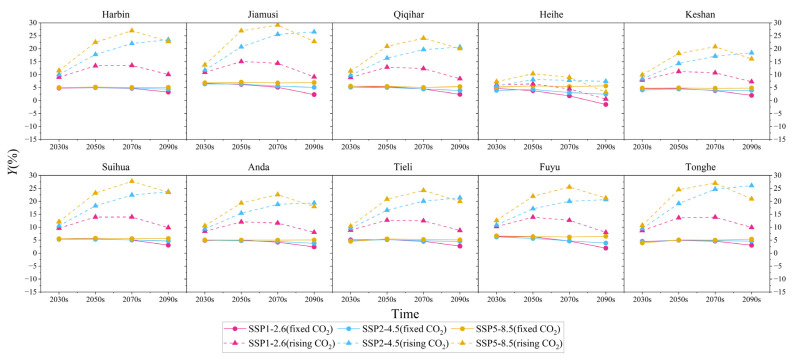
Relative changes in rice yield (*Y*) at each station in the study area from the 2030s to the 2090s under the SSP1-2.6, SSP2-4.5 and SSP5-8.5 scenarios.

Relative changes in yield under the different treatments are shown in Figure 6 and Table 2. At the regional scale, under the rising-CO_2_ treatment, mean yield increases range from 8.87% to 11.02% in the 2030s, increase to 12.53–20.88% in the 2050s, and reach 12.01–23.70% in the 2070s. In the 2090s, the regional mean increase remains high under SSP2-4.5 and SSP5-8.5, at 20.76% and 18.88%, respectively, whereas SSP1-2.6 declines to 8.01%. By contrast, under the fixed-CO_2_ treatment, the regional mean yield increase remains much smaller, ranging from 2.17% to 5.61% across periods and scenarios. At the station scale, Harbin, Jiamusi, Suihua, Fuyu, and Tonghe are generally among the higher-response sites, with 2090s’ increases under the rising-CO_2_ treatment commonly reaching about 20–30% under SSP2-4.5 and SSP5-8.5. In contrast, Heihe remains at the lower end of the station-level response, with increases generally below 10% and approaching zero or slightly negative values in some periods under SSP1-2.6. Overall, yield increases are consistently larger under the rising-CO_2_ treatment than under the fixed-CO_2_ treatment, and the difference between the two treatments becomes larger over time.

#### 2.3.2. Net Contribution of CO_2_ to Yield

**Figure 7:**
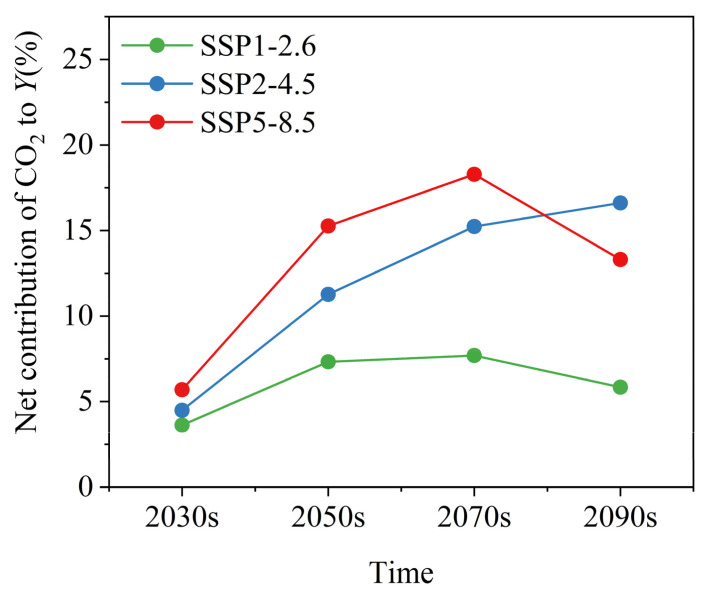
Average net contribution of CO_2_ to rice yield in the study area from the 2030s to the 2090s under the SSP1-2.6, SSP2-4.5 and SSP5-8.5 scenarios.

Figure 7 shows the average net contribution index of CO_2_ to yield under the three SSPs. In the 2030s, the contribution is similar among scenarios, at 4~6%. From the 2050s onward, the scenario differences increase. SSP1-2.6 remains the lowest throughout, with values of 4~8%. SSP2-4.5 shows a continuous increase and reaches about 17% in the 2090s. SSP5-8.5 rises to the highest level in the 2050s and 2070s, peaking at about 18% in the 2070s, and then declines to about 13% in the 2090s.

### 2.4. ET_c_ and Net CO_2_ Contribution

#### 2.4.1. Relative Changes in *ET_c_*

**Figure 8:**
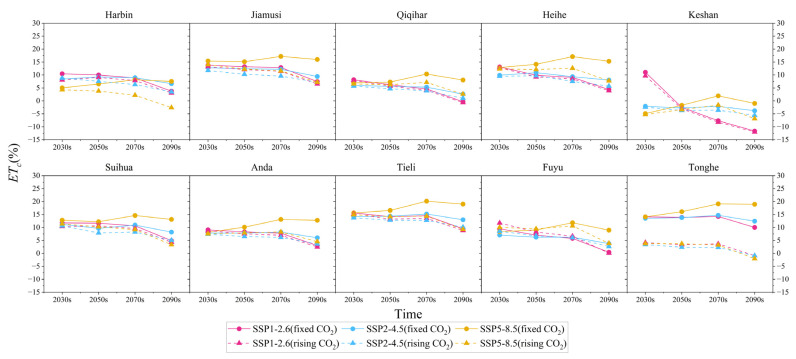
Changes in *ET_c_* at each station in the study area from the 2030s to the 2090s under the SSP1-2.6, SSP2-4.5 and SSP5-8.5 scenarios.

Relative changes in *ET_c_* under the different treatments are shown in Figure 8 and Table 2. At the regional scale, under the rising-CO_2_ treatment, mean *ET_c_* changes decrease over time under three SSP scenarios, from 10.19%, 7.70%, and 7.96% in the 2030s to 1.56%, 3.24%, and 2.70% in the 2090s under SSP1-2.6, SSP2-4.5, and SSP5-8.5, respectively. Under the fixed-CO_2_ treatment, *ET_c_* also declines over time under SSP1-2.6 and SSP2-4.5, from 11.45% and 8.80% in the 2030s to 3.03% and 6.45% in the 2090s, whereas SSP5-8.5 remains at a higher level, increasing from 9.28% in the 2030s to 13.13% in the 2070s and then decreasing slightly to 11.61% in the 2090s. At the station scale, Jiamusi, Tieli, Tonghe, and Suihua generally show relatively high *ET_c_* changes, particularly under the fixed-CO_2_ treatment, with values commonly around 12~20% in the 2050s–2090s. Harbin, Qiqihar, Anda, and Fuyu show intermediate responses, while Keshan differs from the other stations, with *ET_c_* approaching zero or becoming negative from the 2050s onward under most scenarios. Overall, *ET_c_* tends to decline in the 2090s at most stations, but the fixed-CO_2_ treatment, especially under SSP5-8.5, maintains larger *ET_c_* changes than the rising-CO_2_ treatment.

#### 2.4.2. Net Contribution of CO_2_ to *ET_c_*

**Figure 9:**
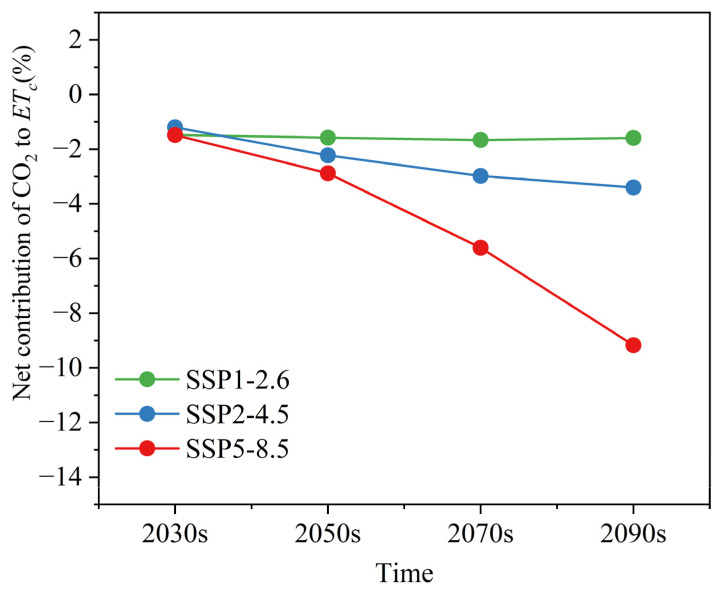
Average net contribution of CO_2_ to *ET_c_* in the study area from the 2030s to the 2090s under the SSP1-2.6, SSP2-4.5 and SSP5-8.5 scenarios.

Figure 9 shows the average net contribution index of CO_2_ to *ET_c_* under the three SSPs. In the 2030s, the values are close among scenarios, at −1~−2%. From the 2050s onward, the differences among scenarios increase. SSP1-2.6 remains nearly stable at −1.5~−1.7%, whereas SSP2-4.5 becomes gradually more negative and reaches about −3.4% in the 2090s. SSP5-8.5 shows the largest decline in magnitude, decreasing from about −1.5% in the 2030s to about −9.1% in the 2090s. These results indicate that the negative contribution of CO_2_ to *ET_c_* strengthens over time, especially under the SSP5-8.5.

### 2.5. WUE and Net CO_2_ Contribution

#### 2.5.1. Relative Changes in *WUE*

**Figure 10:**
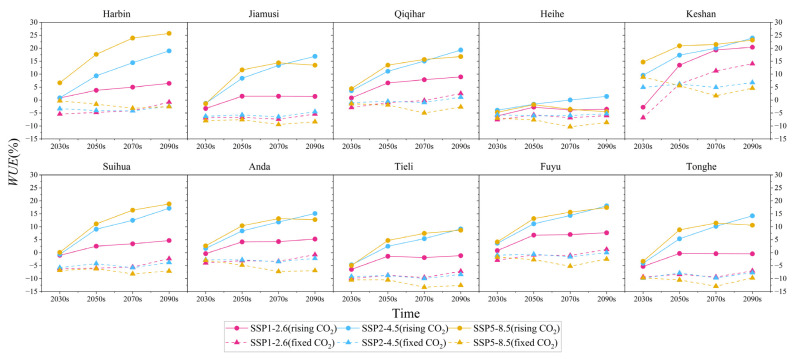
Changes in *WUE* at each station in the study area from the 2030s to the 2090s under the SSP1-2.6, SSP2-4.5 and SSP5-8.5 scenarios.

Relative changes in *WUE* under the different treatments are shown in Figure 10 and Table 2. At the regional scale, under the rising-CO_2_ treatment, mean *WUE* changes increase from −2.28%, 0.47%, and 1.84% in the 2030s to 4.97%, 15.42%, and 14.28% in the 2090s under SSP1-2.6, SSP2-4.5, and SSP5-8.5, respectively. Under the fixed-CO_2_ treatment, by contrast, the regional mean *WUE* remains negative throughout the study period, ranging from −7.59~−1.52% across periods and scenarios. At the station scale, Harbin, Keshan, Suihua, Fuyu, and Tonghe generally show relatively high *WUE* under the rising-CO_2_ treatment, with values in the 2050s–2090s commonly reaching about 10~25%, whereas Heihe remains at a lower level, with values close to zero or below zero during most periods. Under the fixed-CO_2_ treatment, *WUE* at most stations remains near or below zero, while Keshan shows a different pattern and maintains positive values in most periods. Overall, the spatial pattern is broadly consistent across scenarios, but rising CO_2_ leads to higher *WUE* than fixed CO_2_, especially after the 2050s.

#### 2.5.2. Net Contribution of CO_2_ to *WUE*

**Figure 11:**
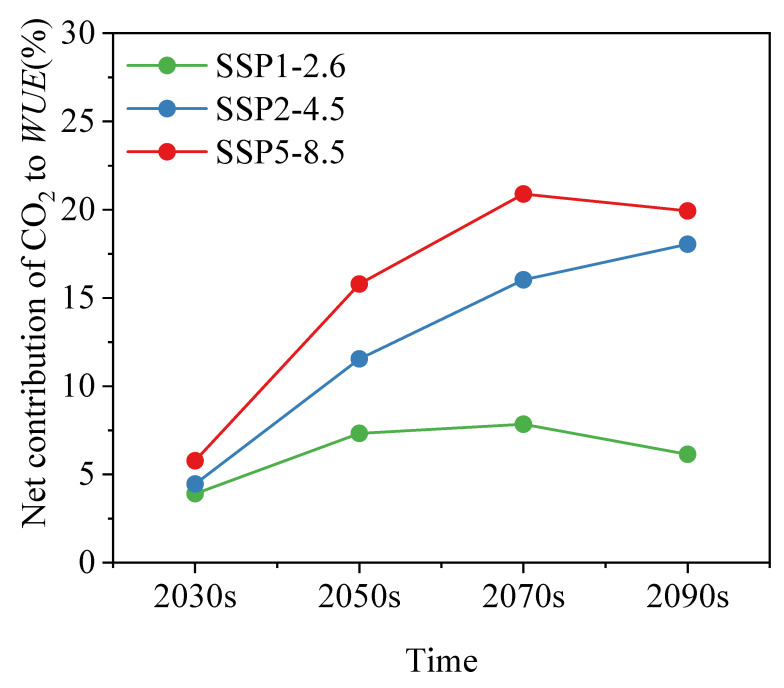
Average net contribution of CO_2_ to *WUE* in the study area from the 2030s to the 2090s under the SSP1-2.6, SSP2-4.5 and SSP5-8.5 scenarios.

Figure 11 shows the average net contribution index of CO_2_ to *WUE* under the three SSP scenarios. In the 2030s, the contribution is similar among scenarios, at 4~6%. From the 2050s onward, the differences among scenarios increase. SSP1-2.6 remains the lowest throughout, increasing only slightly before declining in the 2090s. SSP2-4.5 increases continuously and reaches about 18% in the 2090s. SSP5-8.5 rises more rapidly, peaks at about 21% in the 2070s, and then decreases slightly in the 2090s. These results indicate that the positive contribution of CO_2_ to *WUE* becomes stronger in the 2070s and 2090s, especially under the SSP5-8.5.

### 2.6. Attribution Analysis of Meteorological Factors

**Figure 12:**
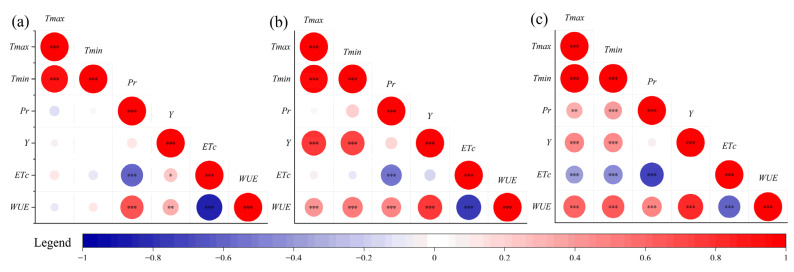
Correlations among meteorological factors, rice *Y*, *ET_c_*, and *WUE* under (**a**) SSP1-2.6, (**b**) SSP2-4.5, and (**c**) SSP5-8.5 (* *p* ≤ 0.05, ** *p* ≤ 0.01, *** *p* ≤ 0.001).

The correlations among meteorological factors, rice yield, *ET_c_*, and *WUE* under the three SSPs are shown in Figure 12. Under SSP1-2.6 (Figure 12a), *T_max_* and *T_min_* are strongly positively correlated with each other. Precipitation (*Pr*) shows a moderate positive correlation with *Y* and *WUE*, whereas *ET_c_* is negatively correlated with *Pr*. The yield (*Y*) has a weak positive correlation with *ET_c_*. *WUE* is strongly negatively correlated with *ET_c_* and moderately positively correlated with *Y*. Under SSP2-4.5 (Figure 12b), *T_max_* and *T_min_* both show positive correlations with *Y*, with coefficients higher than those under SSP1-2.6. *Pr* remains negatively correlated with *ET_c_*, the positive correlation between *WUE* and *T_max_*/*T_min_* becomes stronger, and the negative correlation between *WUE* and *ET_c_* persists. Under SSP5-8.5 (Figure 12c), the positive correlations of *T_max_* and *T_min_* with *Y* become even stronger. The negative correlation between *Pr* and *ET_c_* is also strongest under the SSP5-8.5 scenario. *ET_c_* is negatively correlated with both *T_max_* and *T_min_*, opposite to the pattern in the previous two scenarios. The positive correlation of *WUE* with *T_max_* and *T_min_* is strongest under SSP5-8.5, whereas the strong negative correlation between *WUE* and *ET_c_* remains. Across three scenarios, *T_max_* and *T_min_* consistently show a strong positive correlation with each other, and *WUE* remains strongly negatively correlated with *ET_c_*. It should be noted that the correlation analysis identifies statistical associations among climate variables and simulated indicators; however, it does not establish direct causal relationships.

## 3. Discussion

### 3.1. Yield

In this study, rice yield in Heilongjiang Province generally increased under the rising-CO_2_ treatment across 3 SSPs, whereas yield gains under the fixed-CO_2_ treatment remained much smaller. The yield response was especially pronounced under SSP2-4.5 and SSP5-8.5 after the 2050s, although the increase weakened by the 2090s under SSP5-8.5. This result indicates that future rice yield in the cold-region rice system of Heilongjiang Province may initially benefit from the combined effects of climate change and CO_2_ enrichment, but that the magnitude of this benefit is scenario-dependent and may diminish under stronger rising air temperatures in the 2090s.

Spatial differences among stations may be related to baseline climatic conditions. The stronger yield responses at Harbin, Jiamusi, Suihua, Fuyu, and Tonghe may indicate that moderate warming and elevated CO_2_ partly relieved thermal limitations under their local growing-season conditions. In contrast, Heihe is located farther north, where lower thermal resources and a shorter growing season may limit the benefit of warming. The distinctive *ET_c_* response at Keshan may be associated with local differences in evaporative demand and precipitation conditions. These explanations should be interpreted cautiously because station-level soil and management differences were simplified in the simulations.

The mechanism behind this pattern is likely that rice yield in Heilongjiang Province is still constrained by low growing-season air temperature; therefore, moderate rising air temperatures can relieve thermal limitations, extend effective heat accumulation, and improve biomass formation and grain filling, while elevated CO_2_ further enhances photosynthetic carbon assimilation [32,33]. However, this pattern is not universally reported across rice-growing regions. In tropical and subtropical Asia, rice yield has been shown to exhibit opposing sensitivities to minimum and maximum air temperature [34], with higher nighttime air temperature often reducing yield rather than enhancing it. Likewise, classic field evidence has shown that warmer nights can directly depress rice yield [15], and recent modeling in warm U.S. rice systems projects substantial yield losses under future rising air temperatures because flowering heat stress reduces spikelet fertility [35]. Compared with those warmer systems, the positive yield response found in this study suggests that the thermal background of cold-region rice remains below or near the optimum for much of the century [27,32], so rising air temperatures act less as a direct stressor and more as a partial release from thermal limitations. At the same time, the weakening of yield gain under SSP5-8.5 by the 2090s implies that this cold-region advantage is not unlimited; once rising air temperatures approach or exceed physiological thresholds, rice production in Heilongjiang Province may begin to shift toward the same heat-constrained response reported in warmer rice regions [32,36].

### 3.2. ET_c_

Our study showed that *ET_c_* increased under both treatments, but the increase was consistently smaller under the rising-CO_2_ treatment than under the fixed-CO_2_ treatment, and the negative net contribution index of CO_2_ to *ET_c_* became stronger over time, especially under SSP5-8.5. This indicates that future rising air temperatures alone would tend to enhance *ET_c_* [18], but this effect is substantially offset when rising atmospheric CO_2_ is incorporated into the simulations [18]. In other words, the future *ET_c_* response in Heilongjiang Province is not controlled by air temperature or precipitation alone [18], but by the interaction between climatic forcing and CO_2_-driven physiological regulation [18].

This result can be explained by two concurrent processes. Rising air temperature tends to increase atmospheric evaporative demand, but elevated CO_2_ reduces stomatal conductance and transpiration per unit leaf area [37], while future rising air temperatures may also shorten crop duration and therefore limit cumulative seasonal water consumption [27]. What is important here is that the *ET_c_* response in Heilongjiang Province differs from that reported in hotter or more water-limited rice systems. A recent China-wide assessment projected that elevated CO_2_ generally reduces rice water demand and irrigation requirements [18], but that response is often discussed at the national scale and does not by itself show whether lower *ET_c_* occurs together with stable production or emerges under stress-induced phenological shortening. In arid Egypt, for example, projected *ET_c_* declines over time [37], but that decline is accompanied by strong late-century heat constraints and, without the CO_2_ effect, eventual yield reduction [37]. By contrast, in Heilongjiang Province, the model simulated a smaller increase in *ET_c_* together with higher yield under rising CO_2_, suggesting a potentially more favorable modeled balance between yield gain and crop water consumption under the assumptions used in this study. Therefore, the lower *ET_c_* found here should not be interpreted simply as a universal reduction in crop water use under climate change; rather, in this cold-region rice system it reflects a specific interaction in which CO_2_-induced stomatal regulation suppresses water consumption before heat stress becomes sufficiently strong to dominate the system [32].

### 3.3. WUE

The *WUE* showed the clearest contrast between the two treatments. Under the rising-CO_2_ treatment, *WUE* gradually shifted from slight 2030s changes to substantial increases in the 2050s–2090s, whereas under the fixed-CO_2_ treatment it remained negative throughout the study period. The net contribution index of CO_2_ to *WUE* also increased with time and was strongest under SSP5-8.5. These results suggest that future improvement in *WUE* in Heilongjiang Province depends primarily on rising atmospheric CO_2_ rather than on future rising air temperatures alone.

Mechanistically, this is expected because *WUE* integrates the simultaneous behavior of yield and *ET_c_* [21]. In our study, rising CO_2_ enhanced yield while suppressing *ET_c_* growth, thereby producing positive *WUE* response. However, this outcome is notably different from that reported in several warmer rice systems. In a cold-temperate experimental study, co-elevated CO_2_ and air temperature improved rice *WUE* mainly through increased photosynthetic rate and lower stomatal conductance [33], which is broadly compatible with the mechanism implied here. However, in warm rice regions in the U.S., future climate projections indicate that *WUE* is likely to decline substantially without elevated CO_2_, and even under elevated CO_2_ the mitigation is limited, because yield losses from heat stress remain large [15]. This shows that *WUE* does not respond uniformly to future climate across rice systems. In warm environments, *WUE* may deteriorate because thermal stress suppresses yield faster than CO_2_ can compensate [15,38], whereas in Heilongjiang *WUE* still increases because the CO_2_-induced gain in carbon assimilation and the moderation of *ET_c_* occur under a climate background that remains comparatively favorable for rice growth [33,38]. The net contribution index of CO_2_ to *WUE* under SSP5-8.5 weakened slightly in the late century, which further suggests that even this advantage may become less stable once heat stress intensifies. But at least within the projected periods of this study, the cold-region rice system converts rising CO_2_ into a stronger agronomic and hydrological benefit than has been reported for many warmer rice-growing areas.

### 3.4. Limitations

Several limitations should be considered when interpreting the results. First, only three CMIP6 GCMs were used, although they represent different model structures and institutions, they do not cover the full range of CMIP6 climate uncertainty. Second, the GCM outputs were interpolated to station locations, but no explicit statistical bias correction was applied, which may introduce uncertainty into station-scale crop simulations. Third, the analysis was conducted at 10 representative stations rather than on a continuous gridded surface; therefore, spatial heterogeneity outside the selected stations may not be fully captured. Fourth, future simulations assumed static crop management, cultivar characteristics, irrigation settings, and soil parameters; therefore, the results do not evaluate adaptive management options. Fifth, AquaCrop parameterization, including the FACE-based representation of rising-CO_2_ effects on transpiration and water productivity, may not fully represent all local cultivars, nutrient constraints, or soil-fertility limitations. Finally, the present study did not explicitly simulate extreme events such as cold damage, flooding, drought spells, or flowering-stage heat waves, which may strongly affect rice yield in cold-region systems. Future studies should incorporate larger GCM ensembles, bias-corrected climate forcing, gridded simulations, nutrient and soil-fertility constraints, and management adaptation scenarios.

## 4. Materials and Methods

### 4.1. Overview of the Study Area

**Figure 13:**
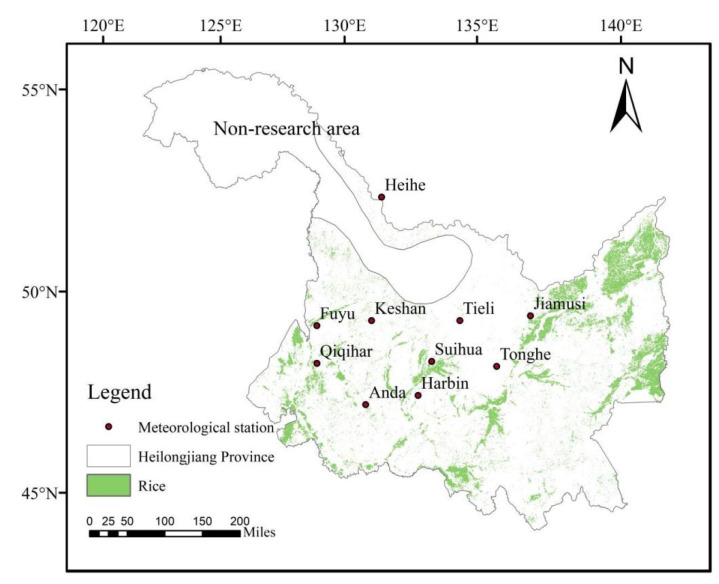
Location of the study area and distribution of the meteorological stations.

The study area is located in northeastern China’s Heilongjiang Province (north latitude 43–53, east longitude 123–135), which is the largest cold-region rice production area in China. Rice is typically transplanted in early to mid-May and harvested in mid-to-late September, with a growing season of approximately 120 to 140 days. The region has a temperate continental monsoon climate, characterized by cool springs and autumns and warm summers. The average air temperature during the rice-growing season (May to September) ranges from 18 to 22 °C. Annual precipitation is about 500 to 600 mm, with June to August accounting for nearly 70% of the crop growth period. Precipitation is highly concentrated and exhibits significant interannual variability [39]. The dominant paddy soils in the study area are black soil, meadow soil, albic soil, with a loam to clay loam and silty clay loam texture. Ten agricultural meteorological stations distributed across the province’s main rice-producing areas were selected as research subjects, covering the major climatic and geographical gradients of Heilongjiang Province: Harbin, Suihua, Anda, Tonghe, Jiamusi, Tieli, Fuyu, Qiqihar, Keshan, and Heihe (Figure 13).

### 4.2. Climate Data

Future climate projections were obtained from the Coupled Model Intercomparison Project Phase 6 (CMIP6). Three global climate models (GCMs) were selected: CanESM5, MPI-ESM1-2-HR, and NorESM2-MM (Table 3) [40]. These models differ in structure, physical parameterization, and climate sensitivity, and their combined use helps represent part of the uncertainty associated with climate-model projections [41]. And this method has been widely used in CMIP6-based agricultural and hydrological impact assessments [40,41]. The models provide projections of key meteorological variables under different Shared Socioeconomic Pathways (SSPs), together with the corresponding greenhouse-gas and CO_2_ concentration changes [42]. To obtain station-scale climate inputs, the GCM outputs were spatially interpolated to the agrometeorological stations using bilinear interpolation. No additional statistical bias correction was applied to the interpolated temperature and precipitation series. The interpolated daily variables included maximum air temperature (*T_max_*), minimum air temperature (*T_min_*), and precipitation (*Pr*). Reference evapotranspiration (*ET*_0_) was then calculated using the FAO Penman–Monteith equation [43].

The three SSPs represent low-, medium-, and high-emission pathways: SSP1-2.6, SSP2-4.5, and SSP5-8.5 [42]. The future simulation period spans 2021–2099 and is divided into four time periods: the 2030s (2021~2040), the 2050s (2041~2060), the 2070s (2061~2080), and the 2090s (2081~2099). Meteorological data for the baseline period (2000–2020) were obtained from observations at the agrometeorological stations in the study area through the China Meteorological Data Service Centre and included daily *T_max_*, *T_min_*, and *P_r_*.

### 4.3. AquaCrop Model

The AquaCrop model (version 7.1), developed by the Food and Agriculture Organization of the United Nations (FAO), was used to simulate rice growth, *ET_c_*, and yield [43]. AquaCrop is a water-driven, process-based crop model that is well suited to evaluating crop yield responses under different environmental and management conditions, especially when water is a key limiting factor [43]. Compared with crop models such as DSSAT and ORYZA, which rely more heavily on detailed ecophysiological processes, AquaCrop uses fewer and more intuitive parameters, thereby balancing simplicity, accuracy, and robustness [41,43]. Since its release, the model has been widely calibrated, validated, and applied to different crops across a range of climatic regions, from field to regional scales, including cold regions [43].

In AquaCrop, crop evapotranspiration is not represented by a single empirical coefficient. Instead, based on the soil–crop–atmosphere continuum, actual evapotranspiration is partitioned into soil evaporation and crop transpiration, and seasonal *ET_c_* is calculated as the sum of daily evapotranspiration over the growing season [43]:

(1) $$\mathrm{ETc}=\sum_{i=1}^{n}(\mathrm{Ei}+\mathrm{Tri})$$

where *E_i_* is soil evaporation on day *i*, *T_r_*_i_ is canopy transpiration on day *i*, and *n* is the number of days in the growing season. This structure distinguishes non-productive soil evaporation from productive crop transpiration and therefore facilitates analysis of the link between yield formation and water use [44].

AquaCrop further links transpiration to aboveground biomass and then simulates final grain yield through the harvest index [44]:

(2) $$B=WP^{*}\times \sum_{i=1}^{n}\left(\frac{{T_{r}}_{i}}{{{ET}_{0}}_{i}}\right)$$

(3) Y=HI·B

where *B* is aboveground biomass (t ha^−1^); *Y* is final grain yield (t ha^−1^); WP* is normalized water productivity, expressed relative to transpiration per unit reference evapotranspiration; *T_r_* is crop transpiration (mm); *ET*_0_ is reference evapotranspiration (mm); and *HI* is the harvest index, that is, the proportion of harvestable product in aboveground biomass. In AquaCrop, *HI* is not fixed but is adjusted dynamically according to water stress, heat stress, and phenological development. Because this framework drives biomass formation through transpiration rather than total evapotranspiration, it can distinguish increased water use from increased productive water use [44].

In AquaCrop, elevated atmospheric CO_2_ influences simulation results through two main pathways. The first is regulation of canopy transpiration: elevated CO_2_ reduces transpiration per unit leaf area by lowering stomatal conductance. Based on FACE experiments, AquaCrop assumes that when atmospheric CO_2_ rises from 369.41 ppm to 550 ppm, the maximum transpiration coefficient declines by about 5% on average [45]. The correction is calculated as follows:

(4) $$K_{c_{T_{r},}x,adj}=K_{c_{T_{r},x}}\left[1-0.05\frac{\mathrm{Ca},i-369.41}{550-369.41}\right]$$

where *Ca*,*i* is the atmospheric CO_2_ concentration in year *i* (ppm). This pathway directly affects transpiration and, consequently, seasonal *ET_c_*.

The second pathway is the adjustment of normalized water productivity (WP*). AquaCrop represents the stimulatory effect of CO_2_ on photosynthetic yield through a correction applied to WP*:

(5) $${\mathrm{WP}}_{\mathrm{adj}}^{*}=\left[1+f_{\mathrm{type}}(f_{{\mathrm{CO}}_{2}-1})\right]{\mathrm{WP}}^{*}$$

where $f_{type}$ is the crop-type correction factor and $f_{{CO}_{2}}$ is the CO_2_ correction factor. For C3 crops such as rice, this effect is generally more pronounced [46]. AquaCrop further weights the CO_2_ correction using a reference concentration, FACE constraints, and sink-strength parameters to capture differences in crop responses to elevated CO_2_. When CO_2_ exceeds 550 ppm, the model also applies additional adjustments to avoid unrealistic extrapolation of gains in WP* under very high concentrations [47]. Thus, in AquaCrop, CO_2_ enrichment does not simply cause a direct increase in yield; instead, it affects yield, *ET_c_*, and *WUE* through the combined effects of reduced transpiration and greater biomass yield per unit transpiration [48,49].

### 4.4. Crop Management, Soil and Irrigation Settings

In this study, simulations used the same crop, soil, and management settings as the calibrated baseline simulations so that projected differences could be attributed to climate forcing and CO_2_ treatment rather than to changes in agronomic management. Soil information for the 10 stations was obtained from the *Harmonized World Soil Database* (HWSD). For each station, the corresponding soil mapping unit was extracted according to the station location. Soil texture information and related soil attributes were then used as inputs to the SPAW Soil Water Characteristics calculator to estimate the main hydraulic parameters required by AquaCrop, including field capacity, permanent wilting point, and saturated hydraulic conductivity. These soil parameters were used to define the soil water balance module in AquaCrop.

Crop calendar settings, including the start and end dates of each growth stage, were derived from historical records for each station. In the simulations, the crop calendar and management settings were kept the same as those used in the baseline simulations. A full-irrigation regime was applied to avoid water stress and to focus on the effects of climate forcing and atmospheric CO_2_ concentration on rice yield, *ET_c_* and *WUE*. Field management was assumed to represent current local management practice, with a bund/ridge height of 0.30 m and optimal weed control. Soil fertility stress was assumed to be non-limiting and was kept constant across all scenarios.

### 4.5. Climate Change Treatment Design

To disentangle the contribution of CO_2_ fertilization from that of other climate drivers, two sets of comparative simulations were designed [47].

In the rising CO_2_ treatment, atmospheric CO_2_ in the model changes over time according to the concentration pathway associated with each SSP, increasing with the emission pathway and future time period [42]. This treatment therefore represents the combined response to climate change and rising-CO_2_.

In the fixed-CO_2_ treatment, atmospheric CO_2_ was held constant at 414.24 ppm. This value corresponds to the CO_2_ concentration in 2020. The 414.24 ppm was used as the baseline-reference concentration at the end of the historical period to isolate the effect of future CO_2_ increase. Through this method, we remove the effect of rising CO_2_ while retaining changes in other climate variables such as air temperature, precipitation, and radiation [47,50]. Both sets of simulations use the same GCM climate forcing; the only difference is the treatment of CO_2_. Comparing the two treatments allows the net contribution index of rising CO_2_ to be quantified [47,50,51].

### 4.6. Calculation of Indicators

Water use efficiency (*WUE*, kg ha^−1^ mm^−1^) is defined as grain yield per unit of crop evapotranspiration and is a key indicator of the relationship between crop yield and agricultural water use. It should be noted that the *ET_c_*-based *WUE* used here differs from transpiration efficiency. Because *ET_c_* includes both soil evaporation and crop transpiration, this indicator is suitable for evaluating crop-scale water productivity and irrigation management implications, whereas transpiration efficiency more directly represents physiological carbon gain per unit of transpired water [37,52]. In agronomic studies and AquaCrop-based applications, *WUE* is commonly expressed as the ratio of yield (*Y*) to crop evapotranspiration (*ET_c_*) [37,52]:

(6) $$\mathrm{WUE}=\frac{Y}{ET_{c}}$$

where *Y* is grain yield (kg ha^−1^) and *ET_c_* is seasonal crop evapotranspiration (mm) during the growing season.

To compare the responses of these indicators under different climate scenarios, changes relative to the baseline period were calculated for rice yield (*Y*), *ET_c_*, and *WUE* as follows:

(7) $$\Delta X=\frac{X_{f}-X_{b}}{X_{b}}\times 100\%$$

where Δ*X* (%) is the percentage change in indicator *X* relative to the baseline period, *X_f_* is the value of the indicator under a future scenario, *X_b_* is the average value during the baseline period, and *X* represents *Y*, *ET_c_*, or *WUE*.

To quantify the effect of rising CO_2_ on rice yield (*Y*), *ET_c_*, and *WUE*, this study used the model-based net CO_2_ contribution index (*C_CO_*_2_), calculated from simulations with rising CO_2_ and fixed CO_2_:

(8) $$C_{CO_{2}}=\frac{X_{rising}-X_{\mathrm{fixed}}}{X_{fixed}}\times 100\%$$

where *X_rising_* and *X_fixed_* represent the simulated rice yield (*Y*), *ET_c_*, or *WUE* under the rising CO_2_ and fixed-CO_2_ treatments, respectively. Positive *C_CO_*_2_ values indicate a positive net contribution index of rising-CO_2_; larger values indicate a stronger relative enhancement due to CO_2_. Because *C_CO_*_2_ is calculated from paired model simulations, it should be interpreted as a model-based scenario-comparison index rather than a strict physical causal attribution.

All changes in yield and *ET_c_* are expressed as percentages relative to the average values during the 2000–2020 baseline period to facilitate comparisons among stations.

### 4.7. Data Processing and Statistical Analysis

Daily meteorological data for the baseline and future periods were first aggregated to the station level and then summarized for the rice-growing season at each station. These data included growing-season *T_max_*, *T_min_*, and *Pr* for the 10 agrometeorological stations. Model outputs of rice yield, *ET_c_*, and *WUE* were then extracted for both the rising-CO_2_ and fixed-CO_2_ treatments. For future climate projections, the arithmetic mean of the three GCMs was used to represent the multi-model ensemble response and to reduce random deviations from any single model [53,54]. Changes in yield and *ET_c_* were expressed relative to the 2000–2020 baseline average, whereas *WUE* and the net contribution index of CO_2_ were calculated using the equations above. Regional averages were obtained by averaging the results across the 10 stations. Statistical analysis focused mainly on descriptive methods to summarize the range and distribution of variables across scenarios, time periods, and stations. Boxplots were used to present the median, interquartile range, and dispersion at the station level, and tables were used to summarize regional-average changes. Data processing and calculations were performed using Microsoft Excel 2019 (Microsoft Corporation, Redmond, WA, USA). Figures and descriptive statistics were generated using OriginPro 2021 (OriginLab Corporation, Northampton, MA, USA), and spatial mapping was conducted using ArcMap 10.8 (Environmental Systems Research Institute, Inc., Redlands, CA, USA).

### 4.8. Model Calibration and Validation

In this study, the AquaCrop model was calibrated using field measurements from agrometeorological stations and irrigation experiment stations across Heilongjiang Province. The data basis, evaluation scale, and sample size used for each variable are summarized in Table S1. The calibration method is manual parameter adjustment. Each station was calibrated separately. A year with near-average precipitation and without major drought or flood anomalies was selected from the recent historical period as the calibration year to minimize the influence of extreme events on parameter estimation. The relevant parameters are listed in Table S2.

Model performance was evaluated using five statistical metrics: the coefficient of determination (R^2^), root mean square error (RMSE), normalized root mean square error (NRMSE), Nash–Sutcliffe efficiency (EF), and the index of agreement (d). Among these, R^2^ measures the linear correlation between simulated and observed values, RMSE reflects the overall magnitude of simulation error, and NRMSE indicates the relative error level; simulation results are generally considered excellent when NRMSE is below 10% [55,56]. EF evaluates model skill relative to the observed mean, with EF = 1 indicating a perfect fit [57]. The index d assesses the overall agreement between simulated and observed values, with values closer to 1 indicating better agreement [58].

(9) $$\mathrm{RMSE}=\sqrt{\frac{1}{n}\sum_{i=1}^{n}{\left(S_{i}-O_{i}\right)}^{2}}$$

(10) $$\mathrm{NRMSE}=\frac{\mathrm{RMSE}}{\bar{O}}\times 100\%$$

(11) $$\mathrm{EF}=1-\frac{\sum_{i=1}^{n}{\left(S_{i}-O_{i}\right)}^{2}}{\sum_{i=1}^{n}{\left(O_{i}-\bar{O}\right)}^{2}}$$

(12) $$d=1-\frac{\sum_{i=1}^{n}{{(O}_{i}-S_{i})}^{2}}{\sum_{i=1}^{n}{\left(\left|S_{i}-\bar{O}\right|+\left|O_{i}-\bar{O}\right|\right)}^{2}}$$

(13) $$R^{2}={\left[\frac{\sum_{i=1}^{n}\left(O_{i}-\bar{O})(S_{i}-\bar{S}\right)}{\sqrt{\sum_{i=1}^{n}{{(O}_{i}-\bar{O})}^{2}}\sqrt{\sum_{i=1}^{n}{{(S}_{i}-\bar{S})}^{2}}}\right]}^{2}$$

In the above equations, *S_i_* and O*_i_* represent the simulated and observed values for the *i*-th sample, $\bar{O}$ is the mean of the observed values, and *n* is the sample size.

## 5. Conclusions

Based on the AquaCrop model and CMIP6 climate projections, this study analyzed projected changes in rice *Y*, *ET_c_*, and *WUE* in Heilongjiang Province under three SSP scenarios (SSP1-2.6, SSP2-4.5, and SSP5-8.5) by comparing simulations under rising CO_2_ and fixed CO_2_. The results show that, under the rising CO_2_ treatment, *Y* increased under all three SSP scenarios and reached its maximum under SSP5-8.5 in the 2070s (23.70%). Under SSP5-8.5, *Y* increased from 11.02% in the 2030s to 23.70% in the 2070s and then declined to 18.88% in the 2090s. Under the fixed CO_2_ treatment, the increase in *Y* remained limited across all scenarios, ranging from 2.17% to 5.61%. *ET_c_* increased in both treatments, but the increase was lower under rising CO_2_ than under fixed CO_2_. For example, under SSP5-8.5 in the 2090s, *ET_c_* increased by 2.70% under rising CO_2_ and by 11.61% under fixed CO_2_. *WUE* increased under rising CO_2_ and reached 15.42% under SSP2-4.5 in the 2090s, whereas under fixed-CO_2_ it remained below the baseline across all scenarios and periods, ranging from −7.59% to -1.52%. These results indicate that the improvement in *WUE* in the cold-region rice system was mainly associated with the increase in *Y* and the lower increase in *ET_c_* under rising-CO_2_. Overall, these results provide useful information for understanding potential climate-change impacts on cold-region rice production and for informing regional adaptation planning. However, because adaptation strategies and management alternatives were not explicitly tested, the findings should be interpreted as scenario-based evidence rather than direct management prescriptions.

## Figures and Tables

**Table 1:** Statistical indicators for AquaCrop model calibration and validation of *ET_c_* and yield.

Index	Period	RMSE	NRMSE (%)	EF	d	R^2^
*ET_c_*	Calibration Period	15.1179	2.9956	0.9944	0.9980	0.9901
	Validation Period	16.1113	3.1764	0.9944	0.9977	0.9905
Yield	Calibration Period	0.3151	4.0849	0.7486	0.9499	0.9410
	Validation Period	0.2096	2.7137	0.8890	0.9727	0.9001

Notes: *ET_c_* validation was based on seasonal total crop evapotranspiration during the rice-growing season, and RMSE for *ET_c_* is expressed in mm. The RMSE for yield is expressed in t ha^−1^. The unit for EF, d and R^2^ is dimensionless.

**Table 2:** Average relative changes in rice *Y*, *ET_c_*, and *WUE* in the study area from the 2030s to the 2090s under the SSP1-2.6, SSP2-4.5 and SSP5-8.5 scenarios under rising-CO_2_ and fixed-CO_2_ treatments.

Period	SSP Scenarios	Projected Change (%)					
		Rising CO_2_			Fixed CO_2_		
		*Y*	*ETc*	*WUE*	*Y*	*ETc*	*WUE*
2030s	SSP1-2.6	8.87	10.19	−2.28	5.24	11.45	−6.33
	SSP2-4.5	9.52	7.70	0.47	5.03	8.80	−4.14
	SSP5-8.5	11.02	7.96	1.84	5.33	9.28	−4.18
2050s	SSP1-2.6	12.53	7.61	3.44	5.20	9.10	−4.04
	SSP2-4.5	16.39	6.49	8.11	5.12	8.56	−3.66
	SSP5-8.5	20.88	7.66	11.01	5.61	10.33	−5.05
2070s	SSP1-2.6	12.01	6.41	4.23	4.32	7.98	−3.86
	SSP2-4.5	19.82	5.93	11.71	4.59	8.78	−4.49
	SSP5-8.5	23.70	7.76	13.60	5.42	13.13	−7.59
2090s	SSP1-2.6	8.01	1.56	4.97	2.17	3.03	−1.52
	SSP2-4.5	20.76	3.24	15.42	4.15	6.45	−2.89
	SSP5-8.5	18.88	2.70	14.28	5.57	11.61	−5.99

Note: *Y*, *ET_c_*, and *WUE* represent rice yield, crop evapotranspiration, and water use efficiency, respectively. All values are expressed as projected changes (%) relative to the baseline period. Under the rising-CO_2_ treatment, atmospheric CO_2_ concentration varies with the SSP scenarios, whereas under the fixed-CO_2_ treatment, atmospheric CO_2_ concentration is held constant at the baseline level.

**Table 3:** Basic information on the selected CMIP6 GCMs.

Climate Model Name	Research Institution	Country	Resolution
CanESM5	Canadian Centre for Climate Modelling and Analysis, Environment and Climate Change Canada	Canada	2.8125° × 2.8125°
MPI-ESM1-2-HR	Max Planck Institute for Meteorology	Germany	0.9375° × 0.9375°
NorESM2-MM	Norwegian Climate Center (NCC)	Norway	2.5° × 1.875°

## Data Availability

Data is contained within the article or the Supplementary Materials.

## Supplementary Materials

The following supporting information can be downloaded at: https://www.mdpi.com/article/10.3390/plants15111625/s1, Table S1: Data basis and sample size used for AquaCrop calibration and validation; Table S2: Reference and calibrated values of key AquaCrop parameters for rice.
