# Supplementary material for: Projected Changes in Yield and Water Use Efficiency of Cold-Region Rice and the Role of CO2 Under Climate Change
Source: Plants (Basel). 2026 May 26;15(11):1625. doi: 10.3390/plants15111625 (PMC13258867; doi:10.3390/plants15111625)
Supplement: Supplementary file 1 [file plants-15-01625-s001.zip › plants-4291169-supplementary.pdf]

Table S1. Data basis and sample size used for AquaCrop calibration and validation.

Variable	Data basis	Spatial coverage	Calibration dataset	Validation dataset	Evaluation scale	Sample size for calibration	Sample size for validation	Metrics
**ETc**	Historical crop water requirement records by growth stage	10 agrometeorological stations	One representative normal year selected for each station	One independent representative normal year selected for each station	Growth-stage cumulative ETc and seasonal ETc	60 growth-stage checkpoints / 10 seasonal values	60 growth-stage checkpoints / 10 seasonal values	RMSE, NRMSE, EF, d, R2
**Yield**	Historical irrigation experiment records and adjusted reference yield under full-irrigation conditions	10 agrometeorological stations	Simulated yield in the calibration year compared with station-level reference yield	Simulated yield in the validation year compared with station-level reference yield	Station-level seasonal grain yield	10 station-level yield pairs	10 station-level yield pairs	RMSE, NRMSE, EF, d, R2

Table S2. Reference and calibrated values of key AquaCrop parameters for rice.

Parameter Category	Parameter	Symbol	Reference Value	Calibrated Value
Threshold air temperatures	Base temperature (°C)	Tbase	8	8
	Upper temperature (°C)	Tupper	30	35
Development of green canopy cover	Initial canopy cover (%)	CC0	3.00-8.00	7
	Canopy growth coefficient（%/day）	CGC	0.006-0.008	0.006
	Maximum canopy cover (%)	CCx	100	85
	Canopy decline coefficient（%/day）	CDC	0.005	0.004
	Minimum effective rooting depth (m)	Zn	0.3	0.25
Development of root zone	Maximum effective rooting depth (m)	Zx	0.6	0.4
Crop transpiration	Crop transpiration	KcTr,x	1.1	1
Crop water productivity	Crop water productivity（g/m^2）	WP*	19	20
Harvest Index	Harvest Index（%）	HI0	35-50	50
Soil water stresses	Soil water depletion threshold for canopy expansion - Upper threshold	pexp,upper	0.4	0.4
	Soil water depletion threshold for canopy expansion - Lower threshold	pexp,lower	0	0
	Soil water depletion threshold for stomatal control - Upper threshold	psto	0.5	0.5
	Soil water depletion threshold for canopy senescence - Upper threshold	psen	0.55	0.5
Plant density	Plant density (plants ha−1)		300,000 – 1,500,000	333000
